# 2073. Persistent HIV-1 Viremia Despite Intensive Antiviral Therapy Due to Non-responsive Clonal Viral Lineages

**DOI:** 10.1093/ofid/ofac492.1695

**Published:** 2022-12-15

**Authors:** Shuntai Zhou, Natalie M Bowman, Claire E Farel, Jessica Lin, Jonathan Parr, David A Wohl, Nathan Long, Julie Nelson, Joseph J Eron, Ronald Swanstrom, Ann Dennis

**Affiliations:** UNC-Chapel Hill, Chapel Hill, North Carolina; University of North Carolina, Chapel Hill, North Carolina; UNC-Chapel Hill, Chapel Hill, North Carolina; UNC-CH, Chapel Hill, North Carolina; UNC-CH, Chapel Hill, North Carolina; University of North Carolina at Chapel Hill School of Medicine, Chapel Hill, North Carolina; UNC-CH, Chapel Hill, North Carolina; UNC-CH, Chapel Hill, North Carolina; University of North Carolina at Chapel Hill School of Medicine, Chapel Hill, North Carolina; UNC-CH, Chapel Hill, North Carolina; UNC-CH, Chapel Hill, North Carolina

## Abstract

**Background:**

After initiation of antiviral therapy (ART), plasma HIV-1 RNA is usually undetectable after one month. In rare cases, viral suppression may not be achieved despite good adherence and with virus susceptible to ART. We used ultra-deep Primer ID next gen sequencing (NGS) to study the viral population and evolution of HIV-1 in two patients with persistent viremia on intensive ART.

**Methods:**

We extracted viral RNA from plasma samples collected at multiple timepoints over the duration of the treatment from two patients (VEX1 and VEX2). We constructed Primer ID NGS libraries covering part of the pol gene and the env V1/V3 region and sequenced them on the Illumina MiSeq platform. We used the ‘tcs’ pipeline to construct template consensus sequences (TCS) for each region, and searched for drug resistance mutations (DRMs). The Geno2pheno pipeline was used to predict co-receptor tropisms.

**Results:**

Patient VEX1 was followed for two years on ART. VEX2 restarted ART in the hospital and received directly observed therapy for nearly 6 months. Both had over 1 million viral copies/mL at the initiation of ART, and the CD4 cell counts were extremely low. Their viral loads slowly declined to approx. 10,000 copies/mL at the end of follow-up but complete viral suppression was not achieved for either patient despite appropriate ART. DRMs were not detected in either patient throughout the treatment with detection sensitivity as low as 0.1%. The sequencing results for VEX1 showed that there were both X4- and R5-tropic viruses at all timepoints and R5 viruses decayed much more slowly than X4 viruses, up to 35-fold more slowly in the initial 3 months of ART. Phylogenetic analysis revealed that the persistent R5 viruses were largely clonal while little clonality was found in the persistent X4 virus. In VEX2, all viruses were R5-tropic. There were three major distinct lineages in the viral population, and two of them completely disappeared after the initiation of ART while the other lineage persisted throughout therapy (Fig 1). The persistent lineage in VEX2 was highly clonal.

Pooled maximum likelihood tree at env V1V3 region of 3 time points from patient VEX2.

**Figure:**
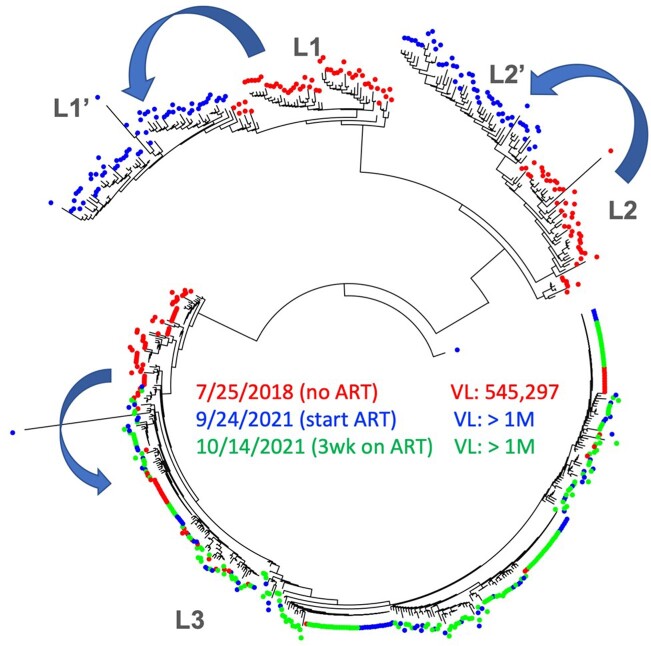

Sequences in red were from 2018 when we obtained the initial specimen from the patient. Sequences in blue were from Sept 2021 when the patient restarted ART. Sequences in green were from Oct 2021 when the patient were on ART for 3 weeks. The patient had 3 major viral lineages, L1, L2, and L3. From 2018 to Sept 2021, we could see the viral evolution of the three lineages (blue arrows). After 3 weeks of ART, L1 and L2 disappeared, while the L3 persisted. We could also see that L3 had several clonal populations which did not respond to ART nor evolve from 2018 to 2021. The additional sequences from Nov 2021 to Feb 2022 were identical to the sequences obtained on Oct 2021 (not shown on this figure).

**Conclusion:**

Our study shows that persistent viremia on ART can come from clonal viral lineages that carry no DRMs. Clonally expanded and infected host cells might contribute to the phenomenon. Further study is needed to explore the origin of these clonal viral genomes.

**Disclosures:**

**Jonathan Parr, MD**, Abbott Laboratories: Donation of laboratory testing and reagents|Gilead Sciences: Grant/Research Support|Virology Education: Honoraria|World Health Organization: Advisor/Consultant|World Health Organization: Grant/Research Support **David A. Wohl, M.D.**, Gilead: Advisor/Consultant|Gilead: Grant/Research Support|Janssen: Advisor/Consultant|Lilly: Grant/Research Support|Merck: Grant/Research Support|ViiV: Advisor/Consultant|ViiV: Grant/Research Support **Joseph J. Eron, MD**, Adagio Therapeutics: data safety monitoring committee|Gilead Sciences: Advisor/Consultant|Gilead Sciences: Grant/Research Support|Glaxo Smith Kline: Advisor/Consultant|Merck: Advisor/Consultant|ViiV Healthcare: Advisor/Consultant|ViiV Healthcare: Grant/Research Support.

